# Antimicrobial Effect of 7-*O*-Butylnaringenin, a Novel Flavonoid, and Various Natural Flavonoids against *Helicobacter pylori* Strains

**DOI:** 10.3390/ijerph10115459

**Published:** 2013-10-28

**Authors:** Sun Hee Moon, Jae Hoon Lee, Kee-Tae Kim, Yong-Sun Park, Seung-Yeol Nah, Dong Uk Ahn, Hyun-Dong Paik

**Affiliations:** 1 Department of Animal Science, Iowa State University, Ames 50011, USA; E-Mails: smoon@iastate.edu (S.H.M.); duahn@iastate.edu (D.U.A.); 2 Division of Animal Life Science, Konkuk University, Seoul 143-701, Korea; E-Mails: jokko77@naver.com (J.H.L.); parkyong@konkuk.ac.kr (Y.-S.P.); synah@konkuk.ac.kr (S.-Y.N.); 3 Bio/Molecular Informatics Center, Konkuk University, Seoul 143-701, Korea; E-Mail: richard44@hanmail.net; 4 Department of Chemistry, Konkuk University, Seoul 143-701, Korea; 5 Department of Physiology, College of Veterinary Medicine, Konkuk University, Seoul 143-701, Korea; 6 Department of Animal Science and Technology, Sunchon National University, Sunchon 540-742, Korea

**Keywords:** flavonoid, *Helicobacter pylori*, 7-*O*-butylnaringenin, hesperitin, antimicrobial effect, urease activity

## Abstract

The antimicrobial effect of a novel flavonoid (7-*O*-butylnaringenin) on *Helicobacter pylori* 26695, 51, and SS1 strains and its inhibitory effect on the urease activity of the strains were evaluated and compared with those of several natural flavonoids. First, various flavonoids were screened for antimicrobial activities using the paper disc diffusion method. Hesperetin and naringenin showed the strongest antimicrobial effects among the natural flavonoids tested, and thus hesperetin and naringenin were selected for comparison with 7-*O*-butylnaringenin. The antimicrobial effect of 7-*O*-butylnaringenin was greater than that of the hesperetin and naringenin. *H. pylori* 51 was more sensitive to 7-*O*-butylnaringenin (2 log reduction of colony forming units, *p* < 0.05) than the other two strains at 200 μM. 7-*O*-Butylnaringenin also showed the highest inhibitory effect against urease activity of *H. pylori*. Morphological changes of *H. pylori* 26695 treated with these flavonoids indicated that both hesperetin and 7-*O*-butylnaringenin at 200 μM damaged the cell membranes.

## 1. Introduction

*Helicobacter pylori* is a micro-aerophilic, Gram-negative, spiral-shaped flagellated bacterium [1]. This microorganism lives in the stomach and duodenum and is known to be one of the most common chronic bacterial pathogens of humans. *H. pylori* is associated with several diseases, including chronic gastritis, peptic ulcers, and gastric mucosa associated lymphoid tissue lymphoma [2,3,4,5]. 

*H. pylori* is resistant to stomach acid because it is protected by the mucous cells and its urease activity [2]. Urease, which is the most characteristic feature of *H. pylori*, catalyzes the hydrolysis of urea to produce ammonia and CO_2_, and protects the bacteria from the acidic environment of the stomach [6,7]. Ammonia itself may cause tissue injury or be toxic to intercellular junctions [8]. However, ammonia generated by the urease buffers gastric acid, and thus is also important for the colonization of the bacteria in the stomach. The urease of *H. pylori* has been described as a highly active enzyme that may be associated with virulence [9] and is considered as a constitutive and permanently active enzyme [10]. The urease in *H. pylori* is a high-molecular weight enzyme that has a high affinity to urea and rapidly hydrolyzes it, but is highly sensitive to urease inhibitors. To treat the patients with gastro-duodenal diseases by *H. pylori*, therefore, inhibiting the infection, growth, and urease activity of *H. pylori* is important. Antimicrobial drugs have been used to treat *H. pylori* infections in recent years, and the successful eradication of this bacterium has been demonstrated to prevent the relapse of duodenal and gastric ulcers [10,11,12]. 

Many naturally occurring compounds found in dietary and medicinal plants, herbs and fruit extracts have been shown to possess antimicrobial activities [13,14,15,16]. Flavonoids are natural compounds ubiquitous in green plant cells [17]. Flavonoids appear to have antimicrobial, antioxidative, anti-inflammatory and anti-carcinogenic effects, and have played major roles in successful medical treatments since ancient times and their use has continued to these days [18,19,20]. There have been various studies on the functional effects of flavonoids with regard to their use by the health food and pharmaceutical industries [21,22,23]. In particular, it has been shown that certain flavonoids have antimicrobial effects against *H. pylori* [13,24,25]. Although the Minimum Inhibitory Concentration (MIC) of some flavonoids against the growth of *H. pylori* has been determined, the nature of the inhibitory effects has not been sufficiently studied [14]. In addition, a new chemically-derived flavonoid has recently been evaluated for its functional activities as a medicinal compound [19]. With this approach, the protective mechanism of some popularly used flavonoids (naringenin and hesperetin), and 7-*O*-butylnaringenin, a new modified flavonoid from naringenin, on the growth as well as the urease activity of *H. pylori* was studied. 

## 2. Experimental Section

### 2.1. Bacterial Strains

*H. pylori* 26695, 51, and SS1 were purchased from the *H. pylori* Korean-Type Culture Collection (HpKTCC, Jinju, Korea). The strains were activated in brucella agar (Difco Laboratories, Detroit, MI, USA) plates supplemented with 5% (v/v) horse serum and was cultured under micro-aerophilic conditions (10% CO_2_ atmosphere) for 3 days. For these studies, the strains were then inoculated in brucella broth supplemented with 5% horse serum and were cultured for 1 day at 37 °C before use.

### 2.2. Flavonoids

**Figure 1:**
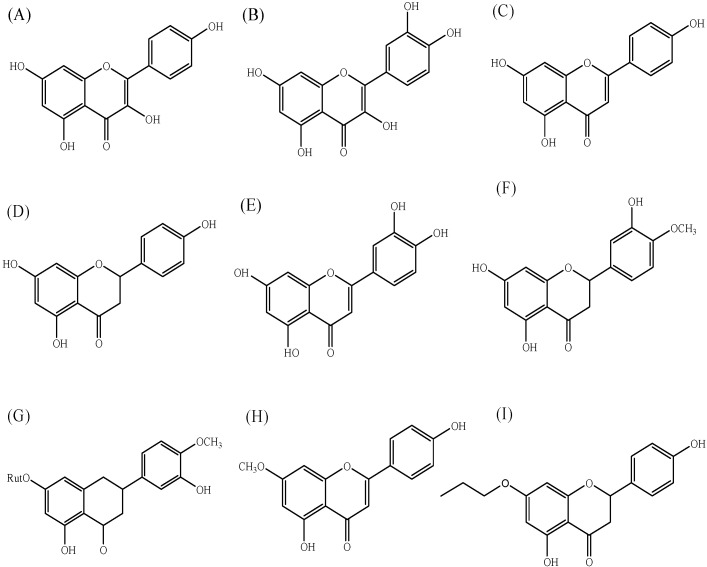
Chemical structures of flavonoids used in this study. (**A**) kaempferol, (**B**) quercetin, (**C**) apigenin, (**D**) naringenin, (**E**) luteolin, (**F**) hesperetin, (**G**) hesperidin, (**H**) genkwanin, and (**I**) 7-*O*-butylnaringenin.

Nine different flavonoids were used for comparison in this study; kaempferol, and quercetin as flavonols, apigenin, luteolin, and 5,4′-dihydroxy-7-methoxyflavone (genkwanin) as flavones, and naringenin, hesperetin, and hesperidin as flavanones [26] (Figure 1). 

All flavonoids were purchased from Sigma Chemical Co. (St. Louis, MO, USA). 7-*O*-Butylnaringenin was supplied by the Chemistry Department in Konkuk University. 7-*O*-Butylnaringenin solution was prepared by mixing and stirring naringenin (500 mg, 1.84 mM), butyl bromide (1.5 equiv.) and K_2_CO_3_ (1.0 equiv.) in dimethylformamide (DMF, 10 mL) for 12 h at 25 °C. The mixture was quenched by adding saturated aqueous NH_4_Cl and then extracted with ethyl acetate. The extracts were then dried over anhydrous MgSO_4_. After evaporation, the resulting crude product was purified using a flash column chromatography, which produced a colorless oil in 35% yield [19]. 

### 2.3. Screening by Paper Disc Diffusion Method

The paper disc diffusion method was used as a screening test for the antibacterial activity of flavonoids [2]. *H. pylori* was incubated as described above. Fourty microliters of flavonoid sample were applied to a paper disc (8 mm in diameter) and the concentrations of flavonoids were 2.5, 5, 10, and 20 mM in dimethylsulfoxide (DMSO), respectively. The DMSO was removed by drying at 20 °C for 10 min, and the paper discs were placed on brucella agar plates supplemented with 5% horse serum inoculated with 2.0 × 10^7^ CFU/mL of each *H. pylori* strain. The zone of inhibition was determined after incubating the plates at 37 °C for 3 days under 10% CO_2_ incubator (MCO-18AIC; Sanyo, Oizumi-Machi, Japan).

### 2.4. Assay of Antimicrobial Effects on *H. pylori*

The initial cell numbers of *H. pylori* strains were adjusted to 2.0 × 10^5^ CFU/mL in broth. Four milliliters of brucella supplemented with 5% (v/v) horse serum, 1 mL of culture broth, and 50 μL of flavonoid solution were added to each well and cultured at 37 °C under 10% CO_2_ atmosphere. The concentration of flavonoid was adjusted to 100 and 200 μM in total broth per well. For the blank and control, 50 μL of distilled water and DMSO were added instead of flavonoid solutions, respectively. After 24 h incubation, culture samples including the blank and control, were serially diluted in 0.1% peptone water and spread on brucella agar supplemented with 5% (v/v) horse serum. Plates were incubated for 3 days at 37 °C under 10% CO_2_ atmosphere [21]. The effect of flavonoids on the *H. pylori* strains was determined using the standard cell counting method. 

### 2.5. Flavonoid Inhibition of Urease in *H. pylori*

Urease activity was measured by the method of Malekzadeh *et al*. [27] and Mobley *et al*. [28]. One thousand eight hundred microliters of urea broth (urea 20 g, NaCl 5 g, KH_2_PO_4_ 2 g, peptone 1 g, glucose 1 g, and phenol red 0.012 g per L solution), 200 µL of the cultured broth, and 20 µL of the flavonoid solutions were mixed in a test tube. The initial cell number of *H. pylori* was adjusted to 2.0 × 10^5^ CFU/mL reaction mixture, and the concentration of flavonoid was adjusted to 200 µM for each reaction mixture. For control, 20 µL of DMSO instead of flavonoid solution was added. After 3 h of incubation at 37 °C, the changes of optical density (pink red color) in urea broth by the ammonia produced were measured at 560 nm with a spectrophotometer (EL311; Bio-Tek Instruments Inc., Seoul, Korea).

### 2.6. Preparation of *H. pylori* for Scanning Electron Microscopy (SEM)

Bacterial cells were prepared for SEM examination using the methods of Tsugawa *et al*. [29]. Cell broth was cultured in brucella broth supplemented with 5% horse serum for 24 h at 37 °C under 10% CO_2_ and were treated with 200 μM of hesperetin, naringenin, or 7-*O*-butylnaringenin. The cells were harvested by centrifugation at 10,000 × g for 10 min and fixed overnight with 25% (v/v) glutaraldehyde at 4 °C. The cells were then washed with 0.1 M phosphate buffer (pH 7.0) three times and dehydrated by washing with ethanol. The ethanol concentration for washing was gradually increased from 10% to 100% in a sequential manner. The dehydrated specimens were treated with ion-spotter-coating at 7 V and 15 mA in a vacuum evaporator (E-1010, Hitachi Science System Ltd., Hitachinaka, Japan) and subjected to SEM (S-3000N, Hitachi Science System Ltd.).

### 2.7. Statistical Analysis

All results are presented as mean ± standard error (SE), and statistical analysis was performed using the SPSS (Chicago, IL, USA) package for Windows (Ver. 18.0). The mean values were compared using one-way analysis of variance (ANOVA) followed by Duncan’s multiple range tests with a significance defined at *p* < 0.05. Correlations were determined using Pearson’s correlation coefficient.

## 3. Results and Discussion

### 3.1. Screening by Paper Disc Diffusion Method

Quercetin, kaempferol, naringenin, and hesperetin had stronger antimicrobial effects than the other flavonoids tested. The antimicrobial effects of naringenin and hesperetin were greater than those of other flavonoids used in this study. Apigenin, hesperidin, and genkwanin had no effects, even at 20 mM, against all *H. pylori* strains studied (Table 1). 

**Table 1:** Zone of growth inhibition of *H. pylori* 26695, 51, and SS1.

Flavonoid	Conc. (mM)	*H. pylori* 26695	*H. pylori* 51	*H. pylori* SS1
Kaempferol	2.5	-	-	-
	5	++	-	+
	10	++	+	+
	20	++	+	++
Quercetin	2.5	-	-	-
	5	-	-	-
	10	-	-	+
	20	+	-	+
Apigenin	2.5	-	-	-
	5	-	-	-
	10	-	-	-
	20	-	-	-
Naringenin	2.5	-	-	-
	5	+++	+++	+++
	10	+++	+++	+++
	20	+++	+++	+++
Luteolin	2.5	-	-	-
	5	+	+	-
	10	++	++	+
	20	++	++	+
Hesperetin	2.5	-	-	-
	5	+++	+++	+++
	10	+++	+++	+++
	20	+++	+++	+++
Hesperidin	2.5	-	-	-
	5	-	-	-
	10	-	-	-
	20	-	-	-
Genkwanin	2.5	-	-	-
	5	-	-	-
	10	-	-	-
	20	-	-	-
7-*O*-Butylnaringenin	2.5	-	-	-
	5	-	++	+
	10	+	+	+
	20	++	++	++

-: No inhibition (≤8.9 mm); +: Slight inhibition (9.0–14.9 mm); ++: Moderate inhibition (15–19.9 mm); +++: Strong inhibition (≥20 mm).

In addition, the antimicrobial effects of flavonoids were slightly different among the *H. pylori* strains tested. 

### 3.2. Evaluation of Antimicrobial Effects on *H. pylori* Strains

**Figure 2:**
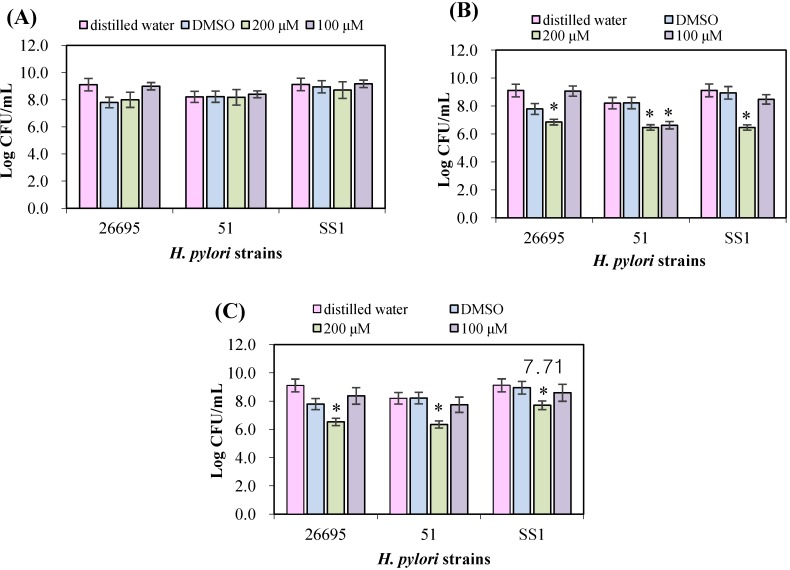
The antimicrobial effects of three flavonoids on growth of *H. pylori* 26695, 51, and SS1. Initial cell density was adjusted to be 2.0 × 10^5^ CFU/mL. (**A**) naringenin, (**B**) hesperetin, and (**C**) 7-*O*-butylnaringenin.

The antimicrobial effects of naringenin, hesperetin, and 7-*O*-butylnaringenin against three *H. pylori* strains indicated that naringenin below 200 mM was the least effective among the three flavonoids and the effect was not significantly different from with blank and control (*p > 0.05*, Figure 2(A)). 

Hesperetin has a stronger antimicrobial effect than naringenin against *H. pylori*, although the effect differed between the strains (Figure 2(B)). Hesperetin (200 μM) showed stronger antimicrobial effects to *H. pylori* SS1 than other two strains and reduced the number by 2.5 log colony forming units (*p < 0.05*). 7-*O*-Butylnaringenin at 100–200 μM showed a stronger antimicrobial effect than the other two flavonoids tested (Figure 2(C)). The antimicrobial effect of 7-*O*-butylnaringenin was >10-fold of that of unmodified naringenin, and 7-*O*-butylnaringenin at 200 μM was highly effective against *H. pylori* 51 (*p < 0.05*). The results indicated that 7-*O*-butylnaringenin was the most effective of all the flavonoids tested at any concentration. However, the effects of 7-*O*-butylnaringenin in the broth culture method were different from those in the disc diffusion method (Table 1). These observations are likely due to the differences in the diffusivity of flavonoids on agar culture and paper disc [22]. The inhibitory effects of flavonoids on the growth of microorganism can vary depending upon the type and size of molecular groups and the positions of their side chains on the flavonoid backbone structure [16,22]. Since many different assays are employed in flavonoid research, including the paper disk diffusion method, the agar dilution assay, the broth microdilution method, the results appear widely conflicting. In particular, assays depending on the diffusion of flavonoids may not give a reliable quantitative measure of antibacterial activity because a strong antibacterial flavonoid may have a low rate of diffusion [23]. 

Values are mean with standard error. * *p* < 0.05 compared with control.

### 3.3. Urease Inhibition by Flavonoids in *H. pylori*

The urease of *H. pylori* is known to be one of the major factors for its resistance to the strong acidic conditions of the stomach [1,7]. *H. pylori* urease produces ammonia from urea and raises the pH of the stomach. Since urea is a small uncharged molecule, it can passively diffuse through the membrane of ureolytic bacteria [2,7]. Naringenin had little effect on the urease activity at <200 μM, while both hesperetin and 7-*O*-butylnaringenin had significant inhibitory effects on the urease activities of three *H. pylori* strains (Table 2). 7-*O*-Butylnaringenin has a stronger inhibitory effect on urease than the other two flavonoids, showing the highest inhibitory effect on *H. pylori* 26695, 51, and SS1 with the inhibition ratios of about 27%, 62%, and 71% at 200 µM concentration, respectively. 

Paulo *et al.* [30] demonstrated that resveratrol and red wines showed an inhibitory effect on *H. pylori* urease activity, which is considered a virulence factor of this organism. Masuda *et al.* [13] reported that gochoonangi (*Wasabia japonica*) has urease inhibitory activity. They showed that the inhibition rates of roots (44.6, 45.8, and 39.7%) and leaves (36.7, 40.2, and 48.7%) on urease activities of *H. pylori* NCTC11637, YS27, and YS50, respectively. Although the anti-*H.pylori* activity of flavonoids has been described by several authors [2,5,6,13,14,16,19,20], there has been little study on the inhibition of urease activity on *H. pylori* strains. 

**Table 2:** The inhibitory effect (%) of various flavonoids at 200 µM on urease activity of *H. pylori* strains after incubation for 3 h at 37 °C.

	Flavonoids	Naringenin	Hesperetin	7-*O*-Butylnaringenin
Strains				
*H. pylori* 26695		1.33 ± 0.13 *^,ax^	0.93 ± 0.15 ^ax^	27.49 ± 0.32 ^bx^
*H. pylori* 51		16.55 ± 1.23 ^ax^	7.31 ± 0.23 ^ax^	61.93 ± 1.45 ^by^
*H. pylori* SS1		13.64 ± 0.98 ^ax^	6.32 ± 0.11 ^ax^	70.75 ± 3.56 ^by^

* Inhibition effect (%) = (OD_control_ − OD_sample_/OD_control_) × 100; ^a–b^ Different letters within a row differ significantly (*p* < 0.05); ^x–y^ Different letters within a column differ significantly (*p* < 0.05).

### 3.4. Morphological Study of *H. pylori* Using SEM

**Figure 3:**
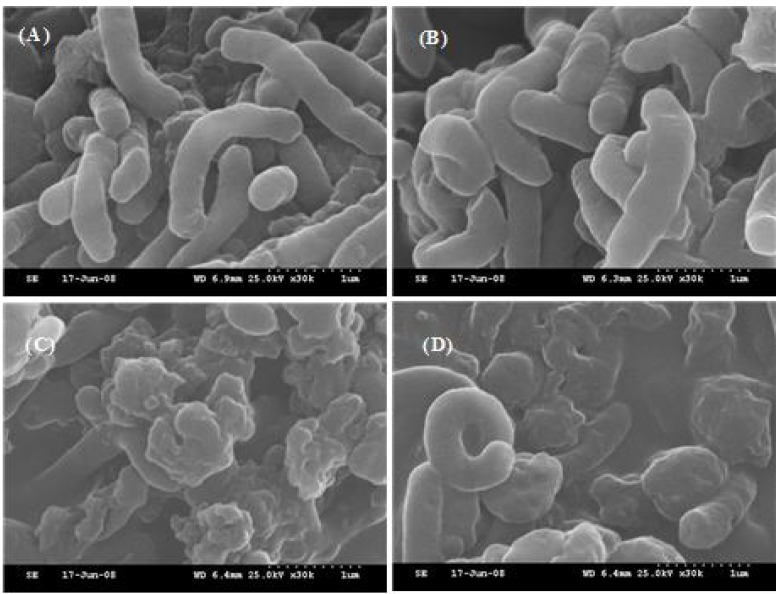
SEM micrographs of *H. pylori* 26695 treated with naringenin, hesperetin, and 7-*O*-butyl naringenin at 200 μM concentration. (**A**) control, (**B**) naringenin, (**C**) hesperetin, and (**D**) 7-*O*-butylnaringenin.

The treatment of *H. pylori* with flavonoids at 200 μM resulted in morphological alterations of the membrane compared with the control (Figure 3). *H. pylori* cells in the control treatment had regular shapes and smooth surfaces (Figure 3(A)), but the cells treated with hesperetin (Figure 3(C)) and 7-*O*-butylnaringenin (Figure 3(D)) at 200 μM were damaged and had irregular shapes and rough surfaces. 

In addition, hesperetin and 7-*O*-butylnaringenin generated different cell damage patterns. Although the morphological changes in 7-*O*-butylnaringenin-treated cells were less pronounced than those with hesperetin-treated cells, 7-*O*-butylnaringenin reduced the viable cell count more and had a stronger inhibitory effect on urease activity than hesperetin (Figure 2 and Table 2). These results indicated that the mechanisms of cell damages may be quite different depending on the kind of flavonoids. On the other hand, naringenin at the same concentration did not cause detectable cell damages (Figure 3(B)). 

The paper disc diffusion results indicated that the cells incubated with hesperetin and 7-*O*-butylnaringenin showed severe membrane damages with membrane integrity disruptions (Table 1). The initial exposure of hesperetin and 7-*O*-butylnaringenin to *H. pylori* caused large surface collapses in the cells and produced wrinkled abnormalities in the cells of *H. pylori* with numerous small clefts, regularly distributed on the bacterial cell surface. Such morphological features in bacterial cells may be due to the lysis of the outer membrane followed by the loss of cellular electron-dense materials on the surface of treated cells [31,32]. Although the mechanisms by which the effects observed in this study are not yet known, it likely involves with the perturbation or inhibition of metabolism such as nucleic acid synthesis, cytoplasmic membrane function, and energy metabolism [22]. 

## 4. Conclusions

In conclusion, the greater inhibitory effect of 7-*O*-butylnaringenin compared with natural naringenin may be helpful in investigating the antimicrobial effects of other flavonoids against *H. pylori*, and in the development of new drugs for patients with gastric diseases caused by *H. pylori*. In addition, 7-*O*-butylnaringenin can be chemically synthesized from orange or tangerine waste, increasing its industrial value as an antimicrobial agent.
